# The Academy for Future Science Faculty: randomized controlled trial of theory-driven coaching to shape development and diversity of early-career scientists

**DOI:** 10.1186/1472-6920-14-160

**Published:** 2014-08-02

**Authors:** Bhoomi K Thakore, Michelle E Naffziger-Hirsch, Jennifer L Richardson, Simon N Williams, Richard McGee,

**Affiliations:** 1 Oakton Community College, Des Plaines, IL 60016, USA; 2 University of Illinois-Chicago, Chicago, IL 60607, USA; 3 Northwestern University Feinberg School of Medicine, 420 E. Superior Ave., Rubloff 647, Chicago, IL 60611, USA

**Keywords:** Biomedical sciences, Biomedical diversity, Graduate students, Intervention, Randomized control trial, Coaching model

## Abstract

**Background:**

Approaches to training biomedical scientists have created a talented research community. However, they have failed to create a professional workforce that includes many racial and ethnic minorities and women in proportion to their representation in the population or in PhD training. This is particularly true at the faculty level. Explanations for the absence of diversity in faculty ranks can be found in social science theories that reveal processes by which individuals develop identities, experiences, and skills required to be seen as legitimate within the profession.

**Methods/Design:**

Using the social science theories of Communities of Practice, Social Cognitive Career Theory, identity formation, and cultural capital, we have developed and are testing a novel coaching-based model to address some of the limitations of previous diversity approaches. This coaching intervention (*The Academy for Future Science Faculty*) includes annual in-person meetings of students and trained faculty Career Coaches, along with ongoing virtual coaching, group meetings and communication. The model is being tested as a randomized controlled trial with two cohorts of biomedical PhD students from across the U.S., one recruited at the start of their PhDs and one nearing completion. Stratification into the experimental and control groups, and to coaching groups within the experimental arms, achieved equal numbers of students by race, ethnicity and gender to the extent possible. A fundamental design element of the *Academy* is to teach and make visible the social science principles which highly influence scientific advancement, as well as acknowledging the extra challenges faced by underrepresented groups working to be seen as legitimate within the scientific communities.

**Discussion:**

The strategy being tested is based upon a novel application of the well-established principles of deploying highly skilled coaches, selected and trained for their ability to develop talents of others. This coaching model is intended to be a complement, rather than a substitute, for traditional mentoring in biomedical research training, and is being tested as such.

## Background

From any perspective other than diversity, the United States has been very successful at developing a talented and creative community of biomedical scientists. However, previous approaches to training have failed to produce satisfactory improvement in the participation of women and individuals from underrepresented minority groups (URMs)^a^, especially beyond the early stages of training. Over the past several decades, the fraction of URMs in faculty and leadership positions has changed little [1,2]. According to the 2010 Census, URMs make up approximately 30% of the U.S. population. However, individuals from these groups only make up approximately 9% of Science, Technology, Engineering and Math (STEM) PhD recipients [3]; National Academy of Sciences, National Academy of Engineering, Institute of Medicine, [4]. Furthermore, only 4% of faculty in basic science departments of U.S. medical schools and biological science departments at the top 50 colleges and universities are URM faculty – a number that has changed little in several decades [1]. This lack of improvement, despite vast infusions of money and the time and energy of many dedicated people, suggests that these efforts are either trying to fix the wrong problem or using the wrong methods to address the problems [3-6]. This lack of diversity in the faculty ranks is the central problem our study is attempting to change. However, we are starting from the time of PhD as the vision and trajectories toward an academic career are critically set during early research training.

Most of the efforts to increase diversity have focused on getting a more diverse pool of young scientists up to the starting line of the PhD (e.g. [7]). These approaches are pursued with the assumption that standard training practices will propel more of them to higher levels of success. However, these standard practices often overlook the very strong social and psychological processes that mediate not only development but perceptions of ability and talent.

Young scientists acquire their research skills under the influences of a sequential series of research mentors through their PhD and postdoctoral training. There is nothing more central to the training of young scientists than a mentor’s guidance. Done right, mentors with adequate skills and time to mentor can have positive impacts on students’ scientific development. At its best, traditional mentoring has the ability to both pass on informal knowledge and allow individuals to “find” other young scientists with mutual interests and develop supportive networks [3,8].

However, the amount and quality of learning and career advice passed on from mentor to mentee can vary widely. Indeed, one of the major limitations of classical mentoring is that it is pedagogically idiosyncratic. Research groups, usually led by a Principal Investigator (PI)/Mentor, in many ways emulate Darwinian natural selection and “survival of the fittest”. The unspoken assumption is that everyone has the same opportunity to develop within the community and therefore the best will “naturally” rise to the top. In theory, mentors provide the informal and formal guidance needed so that those who are the most skilled and independent will thrive. This reliance on informal mentoring is in sharp contrast to many other approaches to development of talent, such as in athletics or the arts. In those fields, rising talent is developed through the guidance of highly skilled professional coaches, leaving less to the often unpredictable elements of mentoring.

A number of programs have focused on improving the quality of mentoring through systematic mentor training [9-12]. Although the findings of these training programs are encouraging, we argue that mentoring as a *construct* is limited for four main reasons. First, mentors face many competing, sometimes incompatible, demands to both produce research for grant renewals and to enable students and postdocs to practice developing novel ideas. Second, good mentoring requires dedicated time, but time has become less available as PIs have to write more grants, lead research teams of increasing size and complexity, and focus on their own survival to support their research groups. Third, PIs constantly face the challenge of assessing the capabilities of different members of their lab group and making judgments of whom to promote to subsequent career stages. These assessments are often subject to unconscious or conscious biases about individual capabilities based on prior educational path and social assumptions based on gender, race, and ethnicity. Fourth, in the informal teaching and learning environment of research groups, a new member who comes from a background that is different from most group members will have a more difficult time figuring out how to excel within the group. As discussed later, we argue that a coaching model can serve to substantially offset these limitations of the traditional mentoring model.

A rare opportunity arose to develop and test our coaching intervention through the American Recovery and Reinvestment Act (ARRA) of 2009. Through an NIH Director’s ARRA Funded Pathfinder Award to Promote Diversity in the Scientific Workforce, we created, “*The Academy for Future Science Faculty*” (referred to henceforth as the *Academy*). The *Academy* recruited established senior life scientists with demonstrated expertise in mentoring young scientists, and provided them with additional training to serve as Coaches in the *Academy*. We recruited two cohorts of PhD students: one just before they started the PhD (*Academy I*) and one near the end of the PhD (*Academy II*). These students were provided with an extensive and unique in-person and virtual coached experience over 2–3 years.

## Methods

### Coaching design and training informed by social science theories

The complexity of the social and cultural factors that impact all young scientists can be understood through the application of social science theories. As will become clear, the social science theories help to explain the lack of improvement in diversity and serve to substantiate the need for a new supplementary coaching model. Our prior research has utilized these social science theories in understanding how students explore and define their science career goals and science identity [13]. We build on these theories by applying them directly to the design of our *Academy* intervention, specifically through the choice and design of the activities and the training of Coaches.

### Communities of Practice

Communities of Practice (CoP) theory derives from the work of Lave and Wenger [14] (see also [15-17]) and helps explain how individuals with common interests and goals work together toward those goals. CoP theory also identifies the processes by which new individuals enter groups and gradually acquire the informal knowledge and practices of the group. According to Wenger [15], the ideal trajectory leads from newcomer to fully accepted and valued member. However, other trajectories can lead to marginalization for individuals who seemingly “do not belong” based on perceptions of lesser competence with the core skills of the group.

PhD programs also function as critical CoPs for incoming students. In U.S. biomedical PhD programs, before students enter their dissertation lab, the program itself strongly informs students’ experiences and helps shape their professional trajectory. But even in program CoPs, URM students can face greater barriers establishing their legitimacy and face greater risks of marginalization [18,19]. Currently, there are a few examples of success in mitigating these issues. The Minority Access to Research Careers (MARC) and the Research Initiative in Scientific Enhancement (RISE) programs (two of the student development programs of the National Institute of General Medical Sciences (NIGMS)) are the entry point for many URM students into the biomedical research training community. These programs help ease entry into research lab CoPs and provide invaluable educational and social capital through program activities. They then serve to broker the transition of students from the undergraduate university community into the PhD training community. The Louis Stokes Alliance for Minority Participation (LSAMP), supported by NSF, plays a similar role in other STEM fields. In many ways, the Program Directors, key faculty, and other program leaders of undergraduate programs like MARC, RISE and LSAMP play the role of coaches and complement what students may or may not get from faculty research mentors.

The movement of a student from an untested newcomer to an accepted member of the lab community can greatly affect that student’s professional future. Yet, it is at this very stage that differences between URM and non-URM members of the group can produce marginalization. More often than not, these non-URM students and faculty mentors are not even aware of this marginalization or are unable to articulate why they are reacting differently, since those reactions stem from unconscious assumptions and biases. With the *Academy*, we argue for the importance and relevance of targeted lectures, workshops and well-established coaching groups to facilitate increased awareness of these issues. Students have opportunities to talk about these CoP challenges and barriers with their coaches and coaching group peers, which in turn creates a supplemental CoP for students to rely on during their graduate school years and beyond.

### Social Cognitive Career Theory

While CoP describes the process by which individuals become part of work groups allowing entry into the community, it does not address the many other variables related to the skill development required of biomedical scientists and the factors mediating career decisions. Any intervention designed to promote success in a particular career must also take into account the developmental processes individuals must undergo to decide to pursue and to be prepared for that career. Several well formulated theories of student development and career evolution describe the process by which students at the individual level transition into becoming scientists and employ their skills in different settings. One of these, Social Cognitive Career Theory (SCCT) has been found by others studying the early scientific development to be particularly useful in understanding their decisions (e.g. [20,21]).

SCCT was established by Brown and Lent [22] and based on the work of Bandura [23] with the primary variables of self-efficacy, outcome expectations, personal goals, and contextual supports/barriers. We are using all of these variables to study the evolution of career interests among young URM and other scientists, but these principles can and are being consciously applied to the new experimental intervention for the development of scientific talent. For example, teaching critical skills such as grant writing can promote self-efficacy as a future PI, and a positive view of the outcome expectation as a future faculty member can be promoted by providing positive role models and tools for dealing with perceived un-achievability of academic careers.

### (Science) identity formation

In our research, the conceptualization of “identity” as it pertains to scientific career development fits well with the reality that identities themselves are not static but constantly being constructed and reconstructed. Further, the various ways in which racial and gender identities can potentially shape individuals’ experiences in new CoPs are important to examine. For our purposes, we are particularly interested in how an “identity-as-scientist” [24] makes an important contribution to persistence within science. Most prior studies and conceptualizations of science identity have focused on undergraduate students, but the continual refinement of identity during graduate training also impacts science career progression.

Studies have shown the critical importance of identity-as-scientist in decisions of individuals to pursue research careers. For example, Chemers et al. [20] and Estrada-Hollenbeck et al. [21] have shown how identity-as-scientist, along with self-efficacy, can predict commitment and persistence toward a science career. Through the *Academy*, we are also interested in learning how racial and gender identities impact individuals’ identities-as-scientists. Another component of our coaching model is therefore its emphasis on *consciously* and *strategically* developing a student’s “identity as a scientist” within a group setting. Coaches also engage students in open and safe discussions about the roles that different identities can play for ‘atypical’ newcomers in new communities of practice.

### Cultural capital

Cultural capital includes any type of useful cultural resource such as knowledge, skills, or behaviors that reside within the individual, or in the form of objects or institutions to which the individual has access. One way scholars have understood social promotion is by examining how cultural capital is valued by different groups [25,26], and how powerful or dominant groups set standards of evaluation based on these values [27]. In general, this theory has been applied extensively in studies of educational inequalities (e.g. [28,29]). Variances in access to the forms of cultural capital valued and rewarded by dominant groups can help account for unequal educational achievement performance in children who originate from different social and class backgrounds [26].

Cultural capital has also been used to understand promotion and fit within the field of science [25,30]. Ovink and Veazey [30] discussed how minority science intervention programs must address not only academics, but also aspects related to the socialization of minority students into the scientific community. The authors argue that such interventions constitute concerted and formal efforts toward expanding the scientific *habitus*^*b*^ which serves to redress minority students’ relative lack of cultural and social capital. Cultural capital in the science field refers to such abilities and resources as networking, bridging cultural expectations between science and the home, interacting with professors, what to say and do during interviews, and how to interact with lab colleagues [30]. Thus, the coaching model attempts to *systematically* create an environment in which cultural capital can be transmitted from experienced Coaches to students, students can exchange cultural capital with each other, and provide both guidance and practice and deploying cultural capital within their PhD training environments.

### The coaching model

An alternative construct to mentoring seldom considered in biomedical research training is based on the type of *coaching* most often seen in the development of talented athletes and artists. As a construct, coaches are highly skilled at teaching, motivating, and developing talents of others (e.g. [31,32]). Career Coaches (to distinguish them from research mentors – hereafter referred to as Coaches) will have the necessary skills for developing the talents of others, but are at an advantage because they do not face the conflicts of interest and time constraints that traditional research mentors often do.

The *Academy* Coaches are not attempting to replace the expertise of research mentors. Rather, they provide complementary focus and expertise to develop student talents. From both their prior experiences and the new theoretical knowledge gained from our trainings, *Academy* Coaches are especially skilled in helping students acknowledge and address some of the extra barriers URM scientists face with each new CoP they enter. From the perspective of the young scientists in this study, these Coaches work to buffer many of the inherent limitations of classical mentoring that we believe play a pivotal role in their professional success and advancement.

Unlike mentoring, coaching as we deploy it emphasizes equally the value and utility of group activities in addition to one-to-one coaching. As such, it seeks to encourage students to learn from one another. Exercises designed to guide professional development, like self-assessment exercises and Individual Development Plans (IDPs), are completed after in-depth and critical discussion in a group setting. Furthermore, *Academy* Coaches are stakeholders in the success of the program, as they all contribute to the development of the coaching curriculum and annual meeting planning. Coaches form a unique coach CoP among themselves to learn from each other and experiment with new approaches to coaching, thus continually improving their coaching expertise.

## Design

### Coach selection

To be an effective Coach, we argue that one must have demonstrated expertise and interest in mentoring, and an intimate knowledge of the requirements and expectations of the discipline or field – in this case, the biomedical research community. Thus, *Academy* Coaches were recruited from among leaders of research training and diversity efforts around the U.S. who had already demonstrated great skills as mentors plus a desire to further develop their skills in guiding others, particularly in diversity goals. In the fall of 2010, announcements advertising the *Academy* were distributed through the Graduate Research Education and Training (GREAT) group of the Association of American Medical Colleges (AAMC) (leaders of PhD and postdoctoral training at U.S. medical schools), and to NIGMS and NSF-funded program directors (e.g. MARC, RISE, Initiative for Maximizing Student Development (IMSD), and Post-baccalaureate Research Education Programs (PREP), LSAMP) to solicit applications for Coaches.

In total, 11 Coaches were selected for *Academy I* (with one serving as an alternate, in case a coach was unable to fulfill their duties for the span of the intervention), and six coaches were selected for *Academy II* (including the one coach originally designated as an *Academy I* alternate). The 16 Coaches come from a range of life science disciplines and a wide range of backgrounds. Demographics of *Academy I* and *Academy II* Coaches are included in Table 1. What all have in common is their previously established record of commitment to diversity through their roles in various diversity efforts.

**Table 1:** **Demographics of *The Academy for Future Science Faculty *** **Career Coaches**

**Coach label**	**Race/Gender**	**Academic rank (at recruitment)**	**Administrative position(s) (at recruitment)**	**Institution**
*Academy I*				
A	White/Male	Professor	Associate Dean	Public Medical School
B	Hispanic/Female	Professor	Program Director	Public Medical School
C	White/Male	Associate Professor	Assistant Dean	Private Medical School
D	Hispanic/Female	Professor	Dean	Private Medical School
E	White/Male	Professor	Director/Senior Associate Dean	Public Medical School
F	White/Female	Professor	Associate Dean/Director	Private Medical School
G	White/Female	Assistant Professor	Associate Dean	Private Medical School
H	Asian/Female	Assistant Professor	Director/Assistant Dean	Private Medical School
I	White/Female	Professor	Associate Dean (Emeritus)	Private Medical School
J	Asian/Male	Professor	Dean/Director	Public Medical School
*Academy II*				
K	Black/Female	Professor	Director	Public Medical School
L	Black/Male	Associate Professor	Assistant Vice-President/ Director	Private Comprehensive University
M	Asian/Male	Professor	Co-Director/Associate Director	Private Medical School
N	White/Female	Professor	Associate Vice-Chancellor/Executive Director	Public Medical School
O	White/Male	Professor	Director	Public Medical School
P	Hispanic/Female	Assistant Professor	--	Private Medical School

### Coaches training

The training of the Coaches for *Academy I* took place over a day and a half in February, 2011. A detailed agenda is included in Additional file 1. The primary elements of this training included:

1. Coaches getting to know each other well – some already knew each other quite well but others did not so it was important to start establishing a Coaches Community

2. An introduction to the four social science theories and models – through lecture and practice looking at research training situations through this theory ‘lenses’

3. Discussing and interpreting the similarities and differences between mentoring which the Coaches were doing extensively and the *Academy* model of coaching

4. Discussing the topics and design elements to include in the first *Academy* meeting to draw on the collective expertise of these very experienced and committed professionals

5. Important confidentially and privacy issued associated with the *Academy* experiment being conducted as an IRB approved randomized controlled trial

The training for the Coaches for *Academy II* took place in February of 2012. It was similar to the training of *Academy I* Coaches but also included more discussion of experiences from *Academy I* to date. It also had to focus on the needs of students nearing the end of their PhDs rather than just starting them as with *Academy I*. The six *Academy II* Coaches were a more diverse group of individuals than those for *Academy I* from the perspective of race, career stage, and institution type.

### Student recruitment

On April 10, 2011, an email describing the *Academy* was sent to biomedical graduate school leaders who were members of the GREAT group, graduate directors at other non-medical major research training universities, and leaders of undergraduate diversity STEM programs throughout the U.S. This date was chosen because it was just before the April 15 common deadline for students to make the final decision of where to pursue their PhDs among the offers of acceptance they had received. Those receiving the emails were asked to forward it to incoming PhD students, or undergraduates they had advised who were starting the PhD in fall 2011. It was advertised as multi-year professional development program particularly designed for those with a strong interest in a future academic career. It was made clear from the start that it was a randomized controlled trial of the program; applicants would not be reviewed, ranked or chosen based on credentials or prior experience. Three hundred and fifty students applied for the *Academy*, representing a wide variety of PhD programs in the U.S. From this applicant pool, 100 students were selected to participate in the *Academy*, and 104 students were selected as study controls who received no intervention. The 204 total students recruited for *Academy I* represents the maximum number of students that could be supported under our funding. Eligibility to participate in the *Academy* required: 1) an expressed interest in an academic career in science; 2) U.S. citizenship or permanent residency; and 3) first year PhD student status.

**Figure 1:**
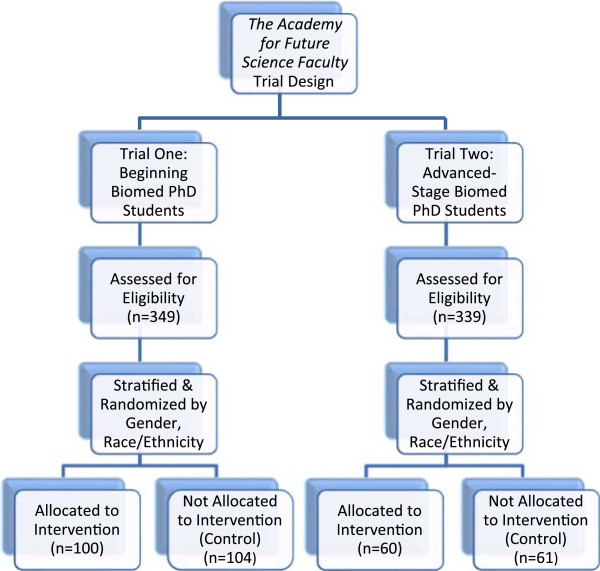
***The Academy for Future Science Faculty *** **trial design.**

In April, 2012, a similar email was sent to the leaders of biomedical PhD training programs describing the second round of the *Academy II*, but this time asking them to forward it to all PhD students who were within 12–18 months of completing their degrees. Approximately 340 applications were received and were stratified and randomized. For this study, 60 were chosen for *Academy II*, and 61 were chosen for controls (to account for anticipated dropouts of the study) (Figure 1). As we anticipated more drop-offs from beginning PhD students compared to those finishing the PhD, the *Academy II* group is smaller than the *Academy I* group.

One of the most critical decisions in designing the *Academy* was whether to include students of all races/ethnicities, or to focus just on URM students. On one hand, it could be argued that an *Academy* for only URM students would concentrate efforts on students who might need these resources most, rather than non-URM students who are more likely to have the resources they need to succeed in the biomedical sciences. However, diversity efforts targeting URM students alone can create a significant stigmatizing effect on those who participate (e.g. [33]). There is also great value in exposing non-URM students to some of the issues and barriers faced by their URM peers as they are discussed in the *Academy* meetings and in the individual coaching groups. Our model also provides opportunities for non-URM students to share inside knowledge that they have gained with their URM peers who may have not yet acquired that knowledge. Finally, the inclusion of both URM and non-URM students was essential in order to determine if the *Academy* differentially could impact URM students vs. all biomedical science PhD students. Therefore, an equal number of men and women were chosen, and equal numbers by race and ethnicity. Thus, for *Academy I*, there are 24 each of Caucasian, Asian, Hispanic and African American students. Only 4 Native American students applied to Academy I, so they were all assigned to the Academy rather than controls as it would not be a meaningful comparison between just 2 students. In *Academy II*, there are 15 each of Hispanic and African American students, 14 each of Asian and Caucasian students, 1 Native American student, and 1 Pacific Islander student. Students in both *Academies* were then assigned to Coaching Groups of 10 students and 1 Coach. These groups were also randomly assigned but stratified such that each group had the maximum diversity possible by gender, race and ethnicity.

### Study design

The *Academy* activities are designed to consciously promote success and advancement while keeping in mind the theory-derived extra challenges URM students can face. For example, the precision and accuracy with which beginning PhD students know what to expect and how to excel in the first PhD year (e.g. cultural capital) is highly variable. Students with less prior research, from less research-intensive universities, and from lower SES backgrounds often can face greater challenges. URM students often face additional identity concerns around fitting in, belonging, finding others like themselves, and the heightened sense of everyone watching their performance. Activities at the *Academy* meetings were designed to bring everyone up to a high understanding of what to expect, acknowledge concerns about fitting in, and assist students to develop strategies for success.

A major focus for both Coaches and the research/program design team was to begin building a CoP among the larger group and individual coaching groups, so they could draw on the collective group expertise and knowledge. Reinforcing the idea that going into research is something that can be consciously and strategically planned and structured, we emphasized that becoming successful as scientists is a learned skill. Through the modeling of a creative and safe environment, another objective and focus of the *Academy* meetings was to encourage students to discuss issues of identity and diversity in science. Students explored their important primary identities, acquired insights into the importance and realities of developing and maintaining identities beyond the scientific identity of a graduate student, and were able to find others within the *Academy* community with whom they shared similar interests and identities. Further, by making the *implicit* process of what students could expect from mentors and PIs more *explicit*, students gained useful cultural capital in the form of insider knowledge about how biomedical science graduate programs function and operate.

One truly unique aspect of the *Academies* was the introduction of the three social science theories through short (15–30 minutes) lectures and discussions of each, showing how the theories translated to practices within the research community. By learning about the underlying, often invisible, social processes within research groups, students (and Coaches) could become more adept at navigating the process of scientific development, ideally preparing them to better fit in within the graduate program and research group CoPs. The *Academy* was therefore developed as a means to create a community of both students who have an expressed desire for a future academic career and successful scientists/professionals with a deep understanding of what is required to achieve and excel in such a career. The *Academy* was designed to encourage a supportive and cohesive community, both within the coaching groups and through the *Academy* as a whole.

### Academy meetings

During the time of the *Academy* funded by the ARRA grant (September, 2010-August, 2013) the *Academy I* meetings consist of a 72 hour in-person meeting in July, 2011 in the Chicago area. Subsequent meetings of both groups lasted 48 hours were held over 3 days. For the meetings in 2012 and 2013, the meeting design included 1 day of overlap between *Academy I* and *II* to allow programming of value to both at the same time plus interactions between the early stage and later stage students. About half of the time at each meeting was devoted for full-group activities and half to Coaching Group time. During these meetings, students were provided with guidance relevant to their upcoming year and stage in graduate school. Every year, students were reminded of the social science theories to help them understand some of the invisible social processes that can affect scientific development. Coaches also reinforced the theories with their groups. The actual topics covered during each meeting were chosen to cover the most critical information and perspectives for the upcoming year, plus longer vision information. For example, in the first *Academy I* meeting, much time was spent on what to expect in the first year of the PhD and how to excel, but a detailed introduction to the road to an academic position was provided. The purpose was to not only facilitate success in the first year but provide a concrete vision of how on achieves an academic career. Equally important in the research design is the introduction of Coaches which is why equal time was spent in full-groups and coaching groups. Time was also scheduled at each meeting for one-to-one meetings between each student and his or her Coach. See Additional file 2 for a sample meeting agenda for *Academy I*, and Additional file 3 for a sample meeting agenda for *Academy II*.

One very unique and novel aspect of the *Academy* meetings was the design and testing of ways to open up meaningful conversations among students and Coaches around issues of privilege, race, bias, and the real-life issues about being ‘different’. This was done subtly during the first 2011 meeting as it was unclear what kind of community would be created when everyone came together. The larger *Academy* community and the Coaching Groups bonded even faster and more strongly than we expected which made it possible to experiment with providing more explicit presentations to create conversations in 2012 and 2013. Those conversations were initiated in 2012 by bringing in an expert in critical race theories and the ongoing influences of racism in America to address the groups and start the conversations. In 2013, a different speaker was brought in to address the psychological and physiological stresses associated with isolation and being ‘the only one,’ and strategies to deal with those stressors. In both cases, the very open, honest, and deep conversations started by these presentations continued on in subsequent Coaching Group discussions and throughout the remainder of each meeting.

### Activities of coaching and coaching groups between Academy in-person meetings

A core element of the *Academy* trial is the ongoing coaching and relationships between the yearly meetings. The need for and value of a Coach is ongoing, although by no means continuous. The *Academy* experiment is providing a variety of tools and approaches to keeping students and Coaches connected throughout the year. This part of the Academy is also experimental as Coaches and students are provided with several options to use as they prefer. The initial design called for Coaching Groups to have monthly or bi-monthly virtual meetings (video and audio) using a product called Adobe Connect. Over time, some groups have migrated to other virtual meeting platforms, some have stopped meeting formally, and others have adopted alternative ways of keeping in touch. Coaches check in with their students on a regular basis via email or phone as well, and students often contact each other independently. Thus, by not rigidly scripting group behavior, each group has been allowed to organically evolve and we are able to gather data on many different variations. A few webinars around specific topics were also delivered to determine how many individuals would find that mode of information delivery of interests.

The initial funding for the *Academy* trial ran out in August, 2013, but a second grant was obtained from NIH to continue some aspects of the study. The new grant enables support of the Coaches and their groups in their virtual meetings, our delivery of new material and experimenting with other ways to provide content, but does not have sufficient funding to enable the annual meeting. However, based on feedback from the last full meetings in 2013, there was a very strong desire to keep meeting in some way. As a result, we have found alternative funding to enable individual Coaching groups to meet, and a majority of them are making plans to do so in the summer of 2014. The new grant also supports the continuation of logistical support for the Coaching Groups, and full collection of data to study how they function, and the comparison between the *Academy* students and the Control. The Coaches, on their own from day one, made it clear to their students that they would be available to them for as long as they desired irrespective of external funding for the *Academy* study.

### Analyses

The primary intention of our intervention is to continue supporting interactions between Coaches and their groups, and follow students longitudinally to see if the *Academy* makes a tangible difference in long-term career choices. The hypothesis is that experimental students will have a higher likelihood of pursuing an academic career after the PhD, and that the proportion of URM and non-URM students will be more equal than the past extremely low rate for URM students. These dynamics will be measured through annual interviews with students and quantitative responses on their perceptions of the achievability, desirability, and their commitment to an academic career throughout graduate school and subsequent career steps. These career steps, such as arranging post-doctoral positions that will likely lead to a successful academic career, will provide interim measures.

### Data collection

Both experimental and control students participate in yearly telephone interviews. Interview question topics include experiences in graduate school, experiences with mentors and PIs, issues of work/life balance and identity, and future career plans. *Academy* experimental students also respond to specific questions regarding their Coaches and the intervention programming. In the past, these interviews took place just before the annual *Academy* meetings or during the summer for Controls. This timing of interviews prior to the individual Coaching group meetings will continue. A sample interview protocol is included in Additional file 4. Prior to their yearly interview, students will complete a pre-interview online survey in which they provide details about fellowships and grants, changes in skill sets, perceptions on the achievability and desirability of an academic career, and current commitment to an academic career. A sample pre-interview survey protocol for *Academy I* is included in Additional file 5. Both *Academy I* and *Academy II* students are asked to discuss their academic experiences over the past year, and to reflect on what will be important for them in the near future. However, *Academy I* students are asked questions related to their early experiences in graduate school, while *Academy II* students are asked questions related to their experiences finishing graduate school and moving on to the next stage of their careers. To compare professional accomplishments as objective measures of possible differences between *Academy* and control students, we intend to obtain a full curriculum vitae from each student about 6 months after graduation.

During *Academy* meetings, daily online surveys obtained evaluative and feedback information about the meeting activities as well as information on impact in real time. Additionally, a virtually complete audio record of all meeting activities was obtained. This included audio recordings throughout the Coaching Group meetings; each Coach was provided with a small digital recorder that was on during all of their discussion. Individual meetings between Coaches and students were NOT recorded, however, to provide a deeper level of privacy to promote open conversations even about delicate topics. *Academy* students also answer interview and survey questions specific to the *Academy* intervention including daily evaluations of the *Academy* meetings in 2011 (Academy I only), 2012, and 2013.

Finally, each of the Coaching Groups was assigned one of our PhD level social scientist Research Associates who observed and took field notes during their meetings. Given that we only had 3 Research Associates and 10 or 6 Coaching Groups, they were unable to be with their groups continuously but rather periodically throughout the meetings.

### Data collected from and about Coaches

As noted above, the goal of this experiment is to study and learn from the Coaches as much as the students. The intent is to take what we learn about various approaches to coaching and synthesize it into a Coaching Institute to train future Coaches. To that end, Coaches completed daily evaluations and were debriefed as a group at the end of each day during *Academy* meetings, Research Associates compiled field notes on the approaches used by their Coaches, and individual interviews were conducted with Coaches after the *Academy* meetings and semi-annually between meetings. A sample interview protocol for *Academy* Coaches is included in Additional file 6. Annual student pre-interview surveys and interviews also asked questions about the students’ interactions with and perceptions of their Coach and Coaching Group.

### Quantitative methods and analysis

We collected quantitative data in the forms of yearly pre-interview surveys and yearly evaluations for the *Academy* in-person meetings. Quantitative data will be analyzed using SPSS version 21 [34]. We will utilize a variety of analyses of variances to determine difference between our sample populations of interest (e.g. control vs. experimental, men vs. women, URM vs. non-URM).

### Qualitative methods and analysis

We collected a variety of qualitative data, including yearly interviews with students and Coaches, and qualitative information from the pre-interview surveys and self-assessments. At the meetings, Research Associates also observed the interactions among Coaches and their coaching group students, and track this information using ethnographic methods such as audio recording and participant observation notes. Qualitative data will be analyzed in NVivo qualitative software [35], and using an interpretation of Grounded Theory as discussed by Coffey and Atkinson [36] see also: [37-39]. Here, Grounded Theory can be seen as being built on abductive, rather than inductive, inference. Interesting themes within the data will be related to broader concepts - including those derived from the broad “stock of knowledge of comparable phenomena” ([36]: 156) of the researchers (including knowledge and experience of *cultural capital*, *identity*, *social cognitive career theory and communities of practice*). However, in keeping with Grounded Theory being an “open-minded intellectual approach”, the analysis will allow for the generation of new (middle-range) theories or “configurations of ideas” ([36]: 156).

### Ethical approval

All research related to the *Academy* was reviewed and accepted by Northwestern University’s Institutional Review Board, Project STU00035424.

## Discussion

The primary aim of this study is to determine the extent to which this theory-derived coaching intervention can address some of the inherent challenges and limitations of traditional research mentoring with respect to progression toward academic careers. Equally important, the trial is designed to study how different successful scientists approach the role of Career Coach, with the ultimate goal of creating a model depicting the critical elements of successful career coaching within the biomedical sciences. Creating the coaching intervention based on social science theories that explain differential acceptance and success within a professional discipline is a truly unique effort to translate theories into practice. The collection and analysis of extensive qualitative and quantitative data throughout the trial will provide an unusually deep and rich data source to understand how it impacts students and coaches.

*Academy* coaching groups were intentionally designed so that no one student is the majority or minority in terms of their race/ethnicity or gender. Additionally, students within coaching groups represent a wide variety of departments, universities, and biomedical subfields. This strategic design has the potential to create a truly objective group in which students can feel free to share their challenges and questions. One potential challenge that students might face with Coaches is the inability to get direct guidance on research, since the Coach’s science expertise may not be in the same area. However, this is not the primary goal of the *Academy*. While students potentially have many resources they can tap into through their department and university, the role of the Coach is to provide the kind of advice and information that is likely unspoken or not readily available. Coaches are also trained to help students determine how to access resources at their home universities.

Future research will use quantitative and qualitative data to examine longitudinal findings among our experimental and control students. In addition to measuring changes in attitudes regarding the achievability, desirability, confidence, and commitment to an academic career, we will examine students’ endeavors as scientists and the extent to which the social science theories inform and are evident and/or in the background in their everyday experiences. Current funding ensures that students and coaches will be followed for at least three more years, which will put Academy I students past graduation into next career steps, and Academy II students well into one or two next career steps. Further, we will be able to determine if *Academy* coaching and programming impacts the decisions among young life scientists to enter academia, and provided them with the tools needed to succeed.

## Endnotes

^a^In our research, we define underrepresented minority groups as those that have been underrepresented in the biomedical sciences – specifically African-Americans, Hispanics, and Native Americans. Asian-American students were considered non-URMs, along with white students, because they are numerically well-represented in science vis-à-vis African-American, Hispanic and Native American students.

^b^Citing Bourdieu, the authors define habitus as “a system of class specific dispositions that shape an individual’s actions so as to reproduce and perpetuate existing systems of hierarchy” (p. 373).

## Abbreviations

URM: Underrepresented minorities; ARRA: American Recovery and Reinvestment Act; NIH: National Institute of Health; SCCT: Social Cognitive Career Theory; CoP: Community of Practice; IDP: Individual Development Plan; GREAT: Graduate Research Education and Training; AAMC: Association of American Medical Colleges; NIGMS: National Institute of General Medical Sciences; NSF: National Science Foundation; MARC: Minority Access to Research Careers; RISE: Research Initiative in Scientific Enhancement; IMSD: Initiative for Maximizing Student Development; PREP: Post-baccalaureate Research Education Programs; LSAMP: Louis Stokes Alliance for Minority Participation.

## Competing interests

The authors state that there are no competing interests.

## Authors’ contributions

RM serves as the Primary Investigator on all grants. All authors contributed to the design, implementation, and data collection of this project. All authors contributed to the writing of this paper. All authors read and approved the final manuscript.

## Pre-publication history

The pre-publication history for this paper can be accessed here:

http://www.biomedcentral.com/1472-6920/14/160/prepub

## Supplementary Material

Additional file 1 The agenda from the 2012 Academy Group 2 Coach Training workshop. Click here for file

Additional file 2 **The agenda from the first *Academy for Future Science Faculty I *** **meeting in 2011.** Click here for file

Additional file 3 **The agenda from the first *Academy for Future Science Faculty II *** **meeting in 2012.** Click here for file

Additional file 4 **Interview protocol for *The Academy for Future Science Faculty I *** **3**^**rd **^**Interview in 2013.** Click here for file

Additional file 5 **Pre-interview survey for *The Academy for Future Science Faculty II *** **2**^**nd **^**interview in 2013.** Click here for file

Additional file 6 **Interview protocol for *The Academy for Future Science Faculty *** **I and II Coaches in Winder 2014.** Click here for file
