# Supplementary material for: The Academy for Future Science Faculty: randomized controlled trial of theory-driven coaching to shape development and diversity of early-career scientists
Source: BMC Med Educ. 2014 Aug 2;14:160. doi: 10.1186/1472-6920-14-160 (PMC4121509; doi:10.1186/1472-6920-14-160)
Supplement: Additional file 1 — The agenda from the 2012 Academy Group 2 Coach Training workshop. [file 1472-6920-14-160-S1.pdf]

Academy for Future Science Faculty Coaches Meeting
March 12-13, 2012
Agenda

Monday, March 12, 2012	
8:15 am - 9:30 am	**Welcome and Introductions**<br>- Overview and format of the next day and a half<br>- Getting to know each other – introduce yourself and provide one tidbit about you that is NOT on your CV<br>- What are you most looking forward to in the next day and a half? During the whole project? Or, what made you crazy enough to volunteer?
9:30 am – 10:00 am	**Stage Setting**<br>- Short Introductory Presentation - Purposes and scope of the Pathfinder Project<br>- Where are we so far – a brief review of the Academy Cohort 1. How Cohort 2 is similar yet different.<br>- What do we need to accomplish while we are here?<br>- Initial discussion on Coaching vs. Mentoring – we will return to this later in the day
10:00 am – 10:15 am	Break
10:15 am – 12:00 pm	**Overview of Social Science Theories Underpinning the Academy**<br>- Why use multiple social science 'lenses'?<br>- The 'bigger picture': What are the key theoretical questions and problems underpinning our study and how are we to address them?<br>**Theory #1 – Social Cognitive Career Theory (SCCT)**<br>- Introductory presentation (Rick McGee):<br>o SCCT tenets, and how biomedical graduate students experiences can be interpreted via SCCT<br>o Questions about SCCT from Decoder and introduction<br>- Group discussion:<br>o How might SCCT help us understand some of the differences in experiences for URM students and women compared to other students by the time we see them in July?<br>o Examples? Can the coaches think of any examples from their experience where SCCT might be useful to understand a particular issue/problem/situation with advanced stage graduate students they have seen? Discussion of examples prepared by the research team.<br>**Theory #2 – Social Identity Theory**<br>- Introductory presentation (Jennifer Richardson-Stovall):<br>o Social Identity Theory and how it expands SCCT to provide additional insights and issues to carefully consider<br>o Initial questions about Social Identity Theory<br>- Group discussion:<br>o How might Social Identity Theory provide insight into some of the differences in

	experiences for URM students and women compared to other students?<br>o Examples? Can the coaches think of any examples from their experience where Social Identity Theory might be useful to understand a particular issue/problem/situation with advanced graduate students they have seen? Discussion of examples prepared by the research team.<br>**Theory #3 – Cultural Capital**<br>- Introductory presentation (Michelle Naffziger):<br>o Cultural capital theory and understanding URM and women students<br>o How does this perspective complement the others we've seen today?<br>- Group discussion:<br>o What "cultural capital" is most important for advanced PhD students to be able to activate effectively as they prepare for their next step?<br>o How do the tacit and explicit "standards of evaluation" impact students and their positioning for next career steps including both self-perceptions and perceptions of mentors and other faculty?<br>o How might gender, race and ethnicity, and class interact with the activation and evaluation of cultural capital?<br>Examples? Can the coaches think of any examples from their experience where Cultural Capital Theory might be useful to understand a particular issue/problem/situation with advanced graduate students they have seen? Discussion of examples prepared by the research team.
12:00 pm - 1:00 pm	Lunch
1:00 pm – 2:45 pm	**Theory #4 – Communities of Practice (C of P)**<br>- Introductory presentation (Simon Williams):<br>o C of P Theory, how it expands from SCCT, Social Identity Theory, and Cultural Capital Theory and brings in the context of scientific development occurring through engagement within a series of research and other groups<br>- Group discussion:<br>o What are the implications of viewing individual research groups as Communities of Practice?<br>o Examples? Can the coaches think of any examples from their experience where C of P Theory might be useful to understand a particular issue/problem/situation with advanced graduate students they have seen? Discussion of examples prepared by the research team.<br>**Integrating the Theories and Beginning to Apply them to the PhD, Postdoc and Career Experiences**<br>- Activity: On your own, take a few minutes to read through the case studies as they are handed out. As you read through them, consider the following:<br>o What are the main challenges facing students during the latter stages of their PhD?<br>o How can we use the four social science theories to better understand the issues/problems/situations detailed in these case studies?<br>o How might you advise or support the student, in light of your experience, but also particularly in light of your new engagement with these social science theories?<br>- Group discussion of cases

	- "One Minute Reflections": on notecards please jot any questions, concerns, ah ha moments, or ideas
2:45 pm - 3:00 pm	Break
3:00 pm - 4:30 pm	**Building a Coaching Paradigm: Principles and Core Elements**<br>- Presentation:<br>o Where are students 'coming from' in terms of their varied experiences during the PhD?<br>o What can/should we coach them toward with respect to knowledge, skills, attitudes, perspectives?<br>- Building Consensus of Primary Objectives for Academy 2<br>o Group Discussion<br>- What are the core components/challenges of coaching?<br>o Consciously designed long-term strategy for moving into an academic career<br>o Tactical advice and support<br>o Forming and maintaining trust<br>o Facilitating access to networks, opportunities, etc.<br>o Building a community of practice within the cohort(s): the challenges of group facilitation.<br>o How do we ensure our coaching doesn't conflict with the mentoring they are receiving in their host institutions?<br>o What else?<br>- How can we use our theoretical models to inform our coaching?<br>o For example, from cultural capital: How do we move beyond a 'deficit' model ("filling up" a student with cultural capital) and toward recognizing and activating cultural capital students already have? How do we make the unspoken standards of evaluation visible for all students?
4:30 pm- 5:30pm	**Proactive coaching guided by theoretical frameworks: consciously guiding students toward academic careers**<br>- Presentation and Discussion<br>o Mentoring versus coaching: How do you envision your role and activities as a coach in the Academy compared to your role as a mentor at your home institution?<br>o How do the theories influence your role as mentor and/ or coach?<br>o What are our mental models of the most important ingredients to deciding upon and achieving an academic career?<br>**Where are we at the end of the day?**<br>o What is your level of confusion? Do the theories seem to fit with and explain biomedical research training and how students experience it differently?<br>o How has today's discussion changed your way of thinking about mentoring or coaching URM and women students during the first year of graduate school?
Before or After Dinner	**End of Day Reflections**:<br>o What are your initial reactions to the information you've received so far? Please discuss any initial questions, concerns or issues you are most interested in or excited about.<br>o Do you have any difficulty or confusion with the models and principles that have been set forth?

	o Can you identify any of these theoretical frameworks in your own practice and belief system regarding mentoring and diversity?<br>o Options for providing written, typed and oral (personal recorder or dialogue) will be provided
6:00 pm – 8:30 pm	Dinner TBD
**Tuesday, March 13, 2012**	
8:15 am – 8:30 am	**Feedback on Day 1 Reflections**
8:30 am - 9:30 am	**What objectives and activities should be included in the July meeting?**<br>- Presentation and discussion – seeding the process and dialogue to be continued after we leave<br>o Post-doc applications<br>o The balance of one-on-one versus group coaching<br>o Students contacting other coaches with specific expertise, experiences or perspectives they can benefit from<br>o Should we establish discipline-based groups (and/or other identity/interest-based groups in addition to assigned coaching groups)?
9:30 am - 10:30 am	**What objectives, activities, and data collection might be included during the academic year between summer 'training camps'?**<br>- The year-round role of the coach: What are the realistic expectations for coaches' time?<br>- Discussion about possible webinar topics; would any coaches like to lead a webinar?<br>- Discussion about the social networking/distance communication element of the Academy
10:30 am – 10:45 am	Break
10:45 am – 11:45 am	**Where do we go from here?**<br>- What format and types of interactions will we use between the full group and smaller coaching groups?<br>- How do we see the various groups (e.g. Coaches, All Students, smaller student groups with coaches, smaller student groups without coaches) as potential Communities of Practice and mediators of the social theories?<br>- What kinds of research and outcome data will we be gathering and studying? Role of the research team in all of the activities discussed above.<br>- Discussion about how the coaches report to the research team on interactions with their students.<br>- How will the first group of 100 students and 10 coaches interact with the second group of 60 advanced students and 6 new coaches – overlap session(s) and contacts after the summer?<br>- What problems or complications should we prepare for or be concerned about?<br>- What level and type of contribution do each of you want to make before July 21?<br>- What else?
11:45 am – 1:00 pm	**Lunch / Follow up Survey Monkey Feedback, Evaluation and Research Data**
