# Supplementary material for: The Academy for Future Science Faculty: randomized controlled trial of theory-driven coaching to shape development and diversity of early-career scientists
Source: BMC Med Educ. 2014 Aug 2;14:160. doi: 10.1186/1472-6920-14-160 (PMC4121509; doi:10.1186/1472-6920-14-160)
Supplement: Additional file 2 — The agenda from the first Academy for Future Science Faculty I meeting in 2011. [file 1472-6920-14-160-S2.pdf]

NORTHWESTERN UNIVERSITY
FEINBERG
SCHOOL OF MEDICINE

# AGENDA

Academy for Future Science Faculty

## Academy for Future Science Faculty

**July 25-28, 2011 - Chicago, IL**

---

**Monday**

**3:30 – 5:15pm**

**Chicago Room**

## Introduction

1. **Welcome to the Academy – Getting Started (3:30 - 4:00pm)**
   - Conceptual framework of the Academy
   - A few ground rules and expectations for developing our community
   - Questions?
   - Informed Consent
     - o If anyone would like to ask private questions about consent before signing, please see Rick McGee
2. **Getting to know each other (4:00 - 5:00pm)**
   - Introducing the coaches and research team
   - Who are you?
     - o At your tables, take turns filling in the blank for the following statement: "Although I love to do research, sometimes I'd rather be..."
3. **Overview of the next 3 days (5:00 - 5:15pm)**

---

**5:15 - 5:30pm** — **Break**

---

**5:30 - 6:30pm** — **Dinner**

---

**6:30 - 8:00pm**

**Chicago Room**

## Our Multiple Identities

4. **Choose a table with a sign that comes closest to fitting your scientific interests. You will have 30 minutes to get to know others at your table with similar interests.**
5. **Choose a table with a sign that either best describes the type of academic career you currently find most desirable, or would like to talk with others about as a career option (see next page).**
   - Primarily research and a small amount of teaching
   - Primarily research with a broad or interdisciplinary research interest and a small amount of teaching
   - An equal balance between research and teaching
   - Primarily teaching with research being done by/with undergraduates
   - Primarily teaching, especially assisting students who come from educationally or economically disadvantaged backgrounds
6. **Create your own identity – For the next 30 minutes you will have the opportunity to seek out and meet others who share a particular interest or identity. It could be anything from a specialized science field to a professional role or personal identity. Examples might be:**

- An interest in improving science education in the community
- An important religious identity
- A parent
- An important racial/ethnic or gender identity
- An 'older' student
- You name it!

7. **Wrap-up – The importance and challenges of recognizing, developing and balancing multiple identities as your scientific identity grows**
8. **Reflections and Evaluation of Day 1**

---

**Tuesday**
**7:00 – 8:30am**

## Breakfast

---

**8:30 - 10:00am**

**Chicago Room**

## Mentoring and Coaching: Part 1

9. **Exercise 1: What do you hope to get from mentors? (8:30 - 8:55am)**
   - Individually, think about and jot down your answer to the following question: <u>As you look forward, what are you hoping for or anticipating getting from your research mentor during the PhD?</u>
   - First in twos, then in groups by table, discuss what you have written down
   - As a table, come to consensus on six of the most important things you'll get from mentors and write <u>one</u> on each of six hexagons
   - Select one person from each table to work with other tables to organize hexagons into common themes on the Theme Wall
10. **Exercise 2: What will be difficult to get or inconsistent? (8:55 - 9:20am)**
    - Individually, think about and jot down your answer to the following question: <u>As you look forward, what do you anticipate might be difficult to get or be inconsistently provided by mentors to different students?</u>
    - First in twos, then in groups by table, discuss what you have written down
    - As a table come to consensus on six of the most important things you'll get from mentors and write <u>one</u> on each of six hexagons
    - Select one person from each table to work with other tables to organize hexagons into common themes on the Theme Wall – work fast!
11. **What do we see on the wall from Exercise 1? What do we see on the wall from Exercise 2? (9:20 - 9:30am)**
12. **Presentation and Discussion: Mentoring as the central training dogma in biomedical research (9:30 - 10:00am)**
    - Strengths and limitations of mentoring as a process for learning
    - Mentoring as a system by which people learn as opposed to classroom learning

---

**10:00 - 10:30am** **Break**

---

| | |
|---|---|
| **Chicago Room**<br><br>**Coaching Group assigned rooms** | ## Mentoring and Coaching: Part 2<br>**13. Discussion: The vision of coaching in the Academy compared to traditional mentoring in graduate programs (10:30 – 11:00am)**<br>**14. Coaching Groups first meeting (11:00 am – noon)**<br>- Move to assigned table or break-out room and get to know each other |
| **12:00 - 1:00pm** | **Lunch (Individual student/coach meeting #1 -12:45 -1:00)** |

## Adapting to and Excelling in Graduate School: Part 1

**1:00 - 2:45pm**

**Chicago Room to start**

The afternoon Coaching Group discussions will focus on some of the most critical aspects of the transition into PhD training and setting yourself up to excel from the first day of graduate school – the first step toward a successful academic career.

**15. A brief introduction to the many changes you will go through during graduate school, the importance of actively engaging in those changes, and the role that early impressions can make in your future. (1:00 - 1:15pm)**

**Move to Coaching Group assigned rooms**

**16. Adapting to new academic approaches and expectations of graduate school (1:15 - 2:00pm)**

Take 5 minutes on your own to jot down answers to the following 2 questions:

- How do you think graduate school classes and formal teaching situations will be different in graduate school compared to undergraduate years?
- What do you think are going to be your most important strategies for adapting to and doing well in graduate school classes?

Take 5 minutes to discuss your list with one other person in your group. In your full group, spend the next 30 minutes discussing your responses to these two questions, with the goal to create a synthesized list on the supplied Word template. <u>One person will take responsibility for compiling the group's list on their laptop.</u>

**17. Choosing Lab Rotations (2:00 - 2:45pm)**

Take 5 minutes on your own to jot down answers to the following questions:

- What are you looking for when considering possible laboratory rotations? (Consider both scientific and non-science factors.)
- As you seek lab rotations, what situations or approaches to mentoring have you experienced in the past that you specifically hope to find again or avoid?

Take 5 minutes to discuss your list with one other person in your group. In your full group, spend the next 30 minutes discussing your responses to these two questions, with the goal to create a synthesized list on the supplied Word template. <u>One person will take responsibility for compiling the group's list on their laptop.</u>

| | |
|---|---|
| **2:45 - 3:15pm** | **Break (Individual student/coach meeting #2 - 3:00 - 3:15pm)** |

**3:15 - 5:30pm**

**Coaching Group assigned rooms**

## What to Expect in Graduate School: Part 2

**18. Making the most of and excelling in lab rotations or the first months in your PhD lab if you are not doing rotations (3:15 - 4:15pm)**

Once you have chosen a lab rotation, it is up to you to excel and get the most from it. Take 10 minutes on your own to jot down answers to the following 5 questions:

- What do you need to accomplish during lab rotations?
- What do the PI and the lab group need to accomplish during a lab rotation?
- How do you figure out what is expected of you and how the lab operates?
- What contributes most to how you are seen by the PI and others in the group and the impression you make?
- How do you ultimately choose a PhD lab?

Take 5 minutes to discuss your list with one other person in your group. In your full group, spend the next 45 minutes discussing your responses to these two questions, with the goal to create a synthesized list on the supplied Word template. One person will take responsibility for compiling the group's list.

**19. Creating our Coaching Manual (4:15 - 5:15)**

Now that we identified some of the most important issues you may face and what you need to accomplish in the first PhD year, we are ready to turn this information into an explicit document to combine and integrate the thinking and expertise of the Coaches and Coaching Groups. We are calling it a Coaching Manual rather than a Coach's Manual because it will focus on multiple resources for guidance not just the Academy Coaches.

- For this first exercise, only consider the first academic year! We will expand and refine the Coaching Manual over the next 2 years.
- Go back to your list of how graduate school classes and academic expectations may be different in graduate school. Transfer one item to the first column of the Coaching Manual Word template provided.
- For each item, insert short bullet statements on the most important Strategies for addressing the issue. Then insert bullets on the roles of the individuals or groups in each successes column. NOTE: not all items will necessarily include substantial roles for all players!!
- Keep doing this for each item your group thinks is important to include.
- As you complete this section, move on to the other two sections on Identifying Lab Rotations and Excelling in Lab Rotations.
- You do NOT need to accomplish this all before dinner. Additional time will be provided for building out your Coaching Manual after dinner.

**20. Full Group Discussion and Reflection on the afternoon and progress on the Coaching Manual (5:15 - 5:30pm)**

**Return to Chicago Room**

21. **An introduction to the theory of Communities of Practice and how it reveals important considerations along your way to an academic career - or any career for that matter! (5:30 – 5:45pm)**

| | |
|---|---|
| **5:45 - 6:45pm** | **Dinner (Individual student/coach meeting #3 - 6:30-6:45)** |

**6:45 - 8:00pm**

## The Coaching Manual: Round 2

**22. Coaching Groups continue filling in the blocks of their Coaching Manual started in the afternoon.**

**(Individual student/coach meeting #4 – 8:00 - 8:15pm)**

**(Individual student/coach meeting #5 – 8:15 - 8:30pm)**

---

**Wednesday**
**7:00 – 8:30 am**

## Breakfast

---

## Self-assessment and Crafting Your Individual Plan: Part 1

**8:30 - 10:00am**

**Chicago Room**

**Coaching Group Rooms**

**23. Introduction to self-assessment as the starting point for success (8:30 - 8:45 am)**

**24. Using peers and Coaches for calibration and guidance from self-assessment (8:45 - 10:00am)**

The next several hours will be devoted to Coaching Group discussions and guidance from Coaches on the topics covered in the Self-Assessment. For each topic, Coaches will lead discussions from their experiences on how to, first, assess your level of skill or preparation and, second, how one acquires skills in these areas during the first few years of graduate school. This is the time for you to raise questions about your own calibrations. ~30 minutes is allocated for discussion of each topic, but some may take shorter or longer than 30 minutes.

During the discussions you may choose to revisit your entries into your Coaching Manuals if new insights arise. For the time until the break, focus on:

- Academic Preparation, including situations where you are
  - Staying in your field
  - Changing fields
  - Specifically consider the value and roles of study groups
- Research Preparation, including situations where you are
  - Staying in your field
  - Changing fields
  - Consider the highly varied range of prior research among beginning PhD students
- Communication Skills, including situations where you use
  - Informal communication and talking about science and non-science
  - Formal communication in varied settings, such as giving lab group talks, short presentations, talking through a poster

---

| | |
|---|---|
| **10:15 - 10:45am** | **Break (Individual student/coach meeting #6 - 10:30-10:45am)** |

## Self-assessment and Crafting Your Individual Plan: Part 2

Continue the conversations started before the break, focusing on:

| | |
|---|---|
| **10:45am - 12:15pm**<br><br>**Coaching Group Rooms** | - Interpersonal Skills<br>  o Knowing yourself and your personal preferences/comforts<br>  o Tools for knowing yourself – e.g. Meyers-Briggs Type Indicator<br>- Scientific Thinking, including your stage of development for<br>  o Understanding what is known – i.e. in the literature and how to critically assess it<br>  o Proposing experiments to test hypotheses presented to you<br>  o Creating new hypotheses<br>  o Linking ideas that don't necessarily seem obviously linked<br>- Networking, including your comfort with and skills to network<br>  o Within your program<br>  o Within your university<br>  o Within your discipline<br>  o Within the Academy<br>- Coping Skills and Stress Reduction<br>  o What causes you stress?<br>  o How do you define "balance" in your life and how do you achieve it?<br>  o Do you use stress to motivate or keep you focused?<br>  o How do you recognize when you need help from others, and how will you make sure you get it? |
| **12:15 - 1:15pm** | **Lunch (Individual student/coach meeting #7 -1:00 -1:15)** |
| **1:15 - 2:15pm**<br><br>**Coaching Group rooms**<br><br>**2:15 – 3:00**<br><br>**Chicago Room** | **Self-assessment and Crafting Your Individual Plan: Part 3**<br><br>**25.Putting it all together for yourself (1:15 - 2:15pm)**<br><br>A Word template will be provided with which each student can work to create your own initial plan and strategy for maximum professional and scientific development during the first year of the PhD. Coaches will be available for consultation.<br><br>**Next Steps – Tools, Time and Keeping Connected**<br><br>26. **Introduction to Edmodo and other electronic tools for supporting the Academy and coaching groups** |
| **3:00 - 3:30pm** | **Break (Individual student/coach meeting #8 - 2:45 -3:00)** |
| **3:30 - 4:30**<br>**Chicago Room**<br><br>**4:00 – 4:30 pm**<br>**Coaching Group rooms**<br>**4:30 -** | **27. Initial plans for regular contact and other support from the Academy leadership and research team**<br><br>**28.Last meeting of Coaching Groups – Plan how you want to keep in touch during the upcoming year before the next meeting in Chicago**<br><br>**Free Time** |
| **5:30 - 7:00pm** | **Dinner** |
| | **Evening Free (Individual student/coach meetings #9 and #10)** |

| | |
|---|---|
| **Thursday** **7:00 – 8:30 am** | **Breakfast** |

## Academic Careers

**8:30 - 10:00am**

**Chicago Room**

**29. Finding Your Path to an Academic Career**

The pathway to an academic career can appear clear, mystical, impossible, obvious, exciting, anxiety-provoking and much more on any given day. This session will 'deconstruct' how it is achieved so that everyone has a clear idea of where they are headed. Although this goal may seem to be a LONG way off, it is actually right around the corner and the most successful students have their eye on this goal continuously. The Academy leadership and Academic Career Coaches will provide a first pass at this topic with much more to come throughout the next two years.

Most important will be questions from students – anything you want to know, reconciling what you have heard, conflicting information, etc. At least half of this session will be devoted to questions and answers.

| | |
|---|---|
| **10:00 - 10:30am** | **Break – Checkout if you have not done so** |

**10:30 - 11:30am**

## Final Pieces

**30. The socially constructed norms and behaviors of the biomedical research community – sometimes clear, sometimes fuzzy, sometimes consistent, sometimes all over the map... (10:30 - 10:50am)**

To become successful in any field you have to understand the unspoken as much as the spoken assumptions and rules by which the members of the community operate – its culture. You will acquire an understanding of these cultural norms over the next several years but it is important to be aware of why they will seem complicated (and sometimes conflicting) ahead of time.

Historically, the normative behaviors among scientists were left unspoken and largely assumed to be effectively passed along through mentoring. Priority was given to autonomy of PIs, their beliefs and their practices. But experience showed this was inadequate and provided too much latitude. Under the umbrella of Responsible Conduct of Research, expected practices and behaviors are becoming more explicit under the banner of Responsible Conduct of Research.

**31. What happens over the next two years? (10:50 - 11:10am)**

**32. End of program evaluation (11:10 - 11:30am)**

| | |
|---|---|
| **11:30 - 1:00pm** | **Take luggage to buses and Lunch** |
| **12:15 & 1:00pm** | **Buses Depart for Airport** |
