# Supplementary material for: The Academy for Future Science Faculty: randomized controlled trial of theory-driven coaching to shape development and diversity of early-career scientists
Source: BMC Med Educ. 2014 Aug 2;14:160. doi: 10.1186/1472-6920-14-160 (PMC4121509; doi:10.1186/1472-6920-14-160)
Supplement: Additional file 3 — The agenda from the first Academy for Future Science Faculty II meeting in 2012. [file 1472-6920-14-160-S3.pdf]

NORTHWESTERN UNIVERSITY FEINBERG SCHOOL OF MEDICINE

AGENDA

Academy for Future Science Faculty

Academy for Future Science Faculty, Group II

July 21-23, 2012 - Chicago, IL

Saturday

2:00 - 3:30pm

Hughes Auditorium

Introductions and Orientation

- Introductions of Coaches and Academy staff
- The Big Picture of the Academies
- Walk through the Agenda for the next 2 days
- Introduction to the social science theories and principles that explain many of the unspoken processes and 'rules' that influence development as scientists – the theoretical underpinnings of the Academies

---

3:30 - 4:15pm
Lurie Building Atrium

Break – getting to know each other!

4:15 - 4:45pm

Hughes Auditorium

Coaching Session 1: Introduction to the importance and tools for self-assessment, constructing short and long-term plans, and Individual Development Plans (IDP)

- Self-Assessment
- Identifying short and long-term goals starting from the long-term and working backwards
- Creating 1, 3 and 5 year plans and IDPs to achieve success in them
- Homework – Self-Assessment to be accomplished before starting Sunday morning program

---

4:45 - 6:00pm

Breakout Rooms

Coaching Group Meeting 1

- Getting to know your group and Coach
- 2 slide 'elevator' introductions
- Clarification of any questions on homework assignment

6:00 - 7:00pm
Lurie Building Atrium

Joint Social Mixer with Academy I students and Coaches

- Focus on meeting students and Coaches from the OTHER group
- Academy II students – as you near the end of your PhD, what experiences, insights, 'ah ha's' do you have for Academy I students to facilitate their success during the PhD?

7:30pm

Dinner in Coaching groups

**Sunday**<br>**7:30 - 8:30am**<br>**Lurie Building Atrium**	**Continental Breakfast**
**8:30 - 11:30am**	**Joint Academy I and II Coaching Sessions**
**8:30 - 10:00am**<br><br>**Hughes Auditorium**	**Coaching Session 2: The core elements of NIH-style research proposals and using them to guide your research – Research proposals are your friends, not the enemy!**<br>- Presentation and discussion - McGee<br>- Introduction to NIH peer review processes – you must learn to write for reviewers<br>- Decoding the types of research and training funding sources available<br>- A walk through the sections of NIH-style research and fellowship proposals and what each needs to accomplish<br>- The Rhetorical Patterns of Writing NIH-style proposals – the secret wink and handshake!
**10:00 - 10:30am**	**Break**
**10:30 - 11:30am**<br><br>**Hughes Auditorium**	**Coaching Session 3: The roadmaps to academic careers – What a search committee looks for when they review applications for academic positions? Data to predict the future!**<br>- Short presentation, Coaches Panel, Q & A<br>- Start thinking about the ultimate goal now – you seldom succeed by accident in this business<br>- Tenure-track, research intensive university<br>- Non-tenure track, research intensive university – the growing niche<br>- Teaching intensive college/university – including different levels of research expectation<br>- Comprehensive university – a balance<br>- The key intermediate steps, including the importance of postdoctoral choices and what you do with them<br>- Building a professional network of colleagues – starting here<br>- The balance between projects where you are solo or a few collaborators vs. multi-person team projects<br>- Many more things to keep in mind and orchestrate
**11:30am**<br>**Lurie Building Atrium**	**Box lunches to take to coaching groups**

**11:30am - 4:00pm**<br><br>**Breakout Rooms**	**Coaching Group Meeting 2**<br>- Discuss any and/or all of the topics covered in the morning sessions<br>- Individual/paired/group work refining your career plans and IDPs<br>- Start and/or complete 1, 3 and 5 year plans + 2 year IDP
**4:00 – 4:30 pm**	**Break – everyone comes back to the full group**
**4:30 - 5:30pm**<br><br>**Baldwin Auditorium**	**Coaching Session 4: Seeking and Designing Your Postdoctoral Position**<br>- Short presentation, Coaches Panel, and Q & A<br>- Insights and discussions about strategies/choices for postdoc positions<br>- Importance of seeking as high a profile lab as possible if you are seeking a research-intensive faculty career<br>- Staying in the same field vs. changing<br>- Conscious plan to seek and achieve increasing independence<br>- Assessing a PI and lab as a good environment – finding things out<br>- Designing your postdoctoral experience for faculty positions with significant teaching<br>- Getting started in a new postdoc group<br>- How to prepare in advance<br>- Getting started – through the lens of Communities of Practice
**5:30 - 6:45pm**<br><br>**Baldwin Auditorium**	**“Stereotypes, Assumptions, Bias, Difference, Diversity, Discrimination: Realities of the ‘modern’ world of biomedical research communities”– led by Dave Stovall**<br>- Biomedical research as a blended meritocracy and socially constructed system<br>- Realities of how we all form opinions and make decisions on often spurious data filtered through cumulative life experiences<br>- How this can lead to ‘success’ being much less merit and accomplishment-based than we like to acknowledge or recognize<br>- Strategies for succeeding within the visible and invisible world of communities of practice to fit-in and be seen as a key insider while maintaining distinct identities and goals<br>- Contributing to constructing of new norms and ‘realities’
**7:00pm**	**Dinner in Coaching Groups**

**9:00pm**<br>**Grey Seminar Room**<br>**Lurie Building**	**(Optional) Continuing and deepening the discussion on difference and discrimination: A facilitated conversation led by Dr. David Stovall, Associate Professor of Educational Policy Studies, University of Illinois at Chicago**
**Monday**<br>**7:30 - 8:30am**<br>**Lurie Building Atrium**	**Continental Breakfast**<br>check out of hotel before coming to the Academy meeting
**8:30 - 9:15am**<br><br>**Baldwin Auditorium**	**Coaching Session 5: Taking control of your own mentoring – Becoming skilled in the core skills of highly effective mentoring relationships**<br>- Framed around core elements of effective mentoring relationships
**9:15 - 9:45am**	**Coaching Session 6: Choosing among research directions as a postdoc and throughout a career**<br>- Short presentation, Coaches Panel and Q&A<br>- High risk/high reward vs. lower risk/reward<br>- Size of fish and size of pond<br>- Aligning research methods with resources available in different settings with different expectations<br>- Establishing identity within a field – pros and cons of multiple identities<br>- Thinking consciously about creativity as it relates to scientific discovery
**9:45am**	**Break and Move to Coaching Groups**
**9:45am-12:00pm**<br>**Breakout Rooms**	**Coaching Groups**<br>- Each group determines how to spend the last 2 hours<br>- Develop plan for ongoing group meetings/communication over the next 6 months to be reassessed at that time
**12:00 - 1:00pm**<br><br>**Baldwin Auditorium**	**Return to full group**<br>- Box lunches<br>- Final words<br>- Evaluation
