# Supplementary material for: The Academy for Future Science Faculty: randomized controlled trial of theory-driven coaching to shape development and diversity of early-career scientists
Source: BMC Med Educ. 2014 Aug 2;14:160. doi: 10.1186/1472-6920-14-160 (PMC4121509; doi:10.1186/1472-6920-14-160)
Supplement: Additional file 4 — Interview protocol for The Academy for Future Science Faculty I 3rd Interview in 2013. [file 1472-6920-14-160-S4.pdf]

Research Study: Academy for Future Science Faculty
Questions for Group I Students – 3rd interview, Prior to Academy 2013

NORTHWESTERN UNIVERSITY

Rick McGee, PhD – Principal Investigator
Northwestern University, Feinberg School of Medicine
r-mcgee@northwestern.edu   Ph: 312-503-1737

This year we are interested in learning about how your experience in your second year went; what being a scientist means to you; how race, ethnicity, gender, and socio-economic status have influenced your experiences; about your future plans and what you have learned through the Academy that you used this year and how you utilized your coach and/or coaching group.

Second Year Research and Experiences

1. Let's start out with a very quick synopsis of how the second year went for you.

   PROBE: Were there any highlights? Surprises? What has been easiest? What has been most difficult?

2. Have you taken your qualifying exam yet? What is the exam format? How did it go?

3. How is your research going? Have you had an opportunity to present or publish anything this year?

4. Tell me about your dissertation topic: what did you choose and how did you go about choosing your topic? Was it a novel idea that you developed or was it an idea developed and encouraged by your PI?

5. How have things gone in your lab? How do you feel about the lab you chose for your research? Was this a good choice for you? Given an opportunity to start over again, would you choose the same lab or a different one? Why or why not?

6. Describe the makeup of your lab – e.g., number of people, men vs. women, grad students, techs, postdocs, from different countries, etc. How do people get along in your lab? Have there been any problems or conflicts?
7. Are there superstars in your lab? Tell me about them.
8. Are there people who seem to be less integrated in your lab? Tell me about them.
9. What are YOUR relationships like in the lab? At this time, how connected or disconnected do you feel with the people in your lab?

10. Tell me about your relationship with your PI.

11. Do you get guidance directly from your PI or is there another person who provides that to you? If so, what is your relationship like with him or her?

12. To what degree do you actively talk with your PI about what you expect/can provide for each other? At this point, does it seem like you will be able to get the mentoring you will need and like from him or her?

    a) If not, what limitations are you seeing?

    b) Do you see other mentors or resources that could meet these potentially unmet needs, or just provide complementary guidance?

13. How about role models? Over the past year have you come across any new people you are starting to see as role models?

14. What type of feedback have you received this year? Who is providing feedback to you?
15. We have previously spoken about how you have figured out the taken-for-granted, “insider” knowledge of how lab groups work. Did you figure out any new insider knowledge this year? Did any of this come from the Academy?
16. Last year we talked about your strengths and weaknesses. How have those strengths and weaknesses changed? Did you identify any new ones? What did you do to address weaknesses?
17. Were there any ways in which being part of the Academy helped you with any challenges this year?
18. Right now, do you feel like you have the balance you would like in your life? Why?
19. What are the things that have caused you stress in your life and in school over the past year? What do you do to reduce stress? What kinds of communities of support do you have, if any?

Being a Scientist

20. Have your views changed over the past year in any way in terms of what it means to be a scientist? Have your views changed in terms of how you see yourself as a scientist? What about how others see you as a scientist?

Race/ Ethnicity, Gender, SES

As you know, we are learning about how gender, race/ethnicity, and socio-economic status/class impact identity and how you see yourself and how others see you. Reflecting on ONLY this past year:

21. Gender:
    a) Has gender impacted your experiences? If so, how?
    b) Do you think your experiences have been different than others of a different gender?
    c) Has your gender impacted how you are viewed as a scientist?
22. Race/ Ethnicity/ Skin color:
    a) Has skin color impacted your experiences? If so, how?
    b) Do you think your experiences have been different than others of a different racial or ethnic background?
    c) Has your skin color impacted how you are viewed as a scientist?
23. Socioeconomic status:
    a) Have socioeconomic factors impacted your experiences? If so, how?
    b) Do you think your experiences have been different from others of different SES?
    c) Has your SES impacted how you are viewed as a scientist?
24. Over the past year, did you come across any new professors, mentors or PIs who share the same racial/ethnic and/or gender background as you? Did you find yourself relating to them differently than other teachers or mentors? In what ways?
25. Have you felt like you are the ‘only one’ or ’one of the only ones’ over the past year? Has this in any way affected you?
26. Has your participation in the Academy caused you to reflect on or interpret differently your experiences BEFORE starting graduate school, in terms of gender, race/ethnicity or socio-economic status?

Thinking about the Future

We are interested in how your second year has impacted or changed the way you think about your future and if the Academy impacted or changed this.

27. What have you heard from others about academic careers over the past year? Have any messages from faculty, grad students, and post docs been particularly important to you in your thinking about or decision-making on careers?
28. Has your image of yourself as a potential professor changed in this past year? If so, what led to these changes?
29. Now let's revisit your longer term future plans. Are there any changes in where you would like to be in 10-15 years' time – both professionally and personally? What has been responsible for your new plans?
30. What are some of the specific steps that you know you will need to take over the next several years in order to accomplish your career goals? How confident are you that you will achieve this goal?
31. Do you see any new major or difficult barriers, personal or professional, that could deter you from achieving your career goal? How do you plan to overcome or work around these barriers?

Coaches and the Academy:

32. We are really interested in gathering your input about how the Academy and coaching model worked this year and what elements were most effective and what might be changed. Do you have any thoughts or suggestions?
33. After you left the second Academy meeting, what were your expectations of your coach during your second year? Were your expectations met? Were your coach and/or coaching group useful during this past year? In what ways? If you didn't reach out to your coach, why not?
34. Can you tell me about a particularly memorable moment from your interaction with your coach over the past year?
35. Did you ever turn to other Academy students for help with a particular issue this year? Tell me about it.
36. I've read your answers in the survey about whether you've used the social science theories in the past year. There are a few points that I would like you to expand on.
37. This last question is different as we are using this as feedback for coaches - they are interested in continually improving their skills as coaches. What could your coach have done differently to make the process more useful for yourself and/or the group as a whole? Is there anything that got in the way of your coach's effectiveness for you and/or the group?
