# Supplementary material for: The Academy for Future Science Faculty: randomized controlled trial of theory-driven coaching to shape development and diversity of early-career scientists
Source: BMC Med Educ. 2014 Aug 2;14:160. doi: 10.1186/1472-6920-14-160 (PMC4121509; doi:10.1186/1472-6920-14-160)
Supplement: Additional file 5 — Pre-interview survey for The Academy for Future Science Faculty II 2nd interview in 2013. [file 1472-6920-14-160-S5.pdf]

Research Study: The Academy for Future Science Faculty
Survey Prior to the Second Interview, Group II

Rick McGee, PhD – Principal Investigator
Northwestern University, Feinberg School of Medicine
r-mcgee@northwestern.edu 312-503-1737

Please provide the information listed below so we can understand your experiences over the past year and the role that the Academy played in those experiences. All of this information will be kept strictly confidential amongst the Principle Investigator and his team, just like your interviews for the study. No information you provide will ever be revealed in any way that could identify it with you. This information will not be shared with the coaches either. You are free to choose not to answer any of the questions.

First Name* ______________________________ **Last Name***______________________________

*Required

Address ____

Email _________________________________ **Mobile Phone** _______________________________

Coach (Drop down menu w/coach names to choose from)

1. **Marital status** ___single ___married/partnered ___separated/divorced ___widowed
2. If you have children, what are their ages?_______________________________ ___No children
3. What is the title of your PhD dissertation (or tentative title)? (open text)
4. Did you apply for any fellowships or grants this year? If so, please describe. Include mention of any specific skills or insights you used from last year's Academy session on NIH grants, if applicable. (Please indicate N/A if this does not apply) (200 word count) (open text)
5. If you participated in <u>any academic or career-related programs or special activities during the previous year of graduate school</u>, what did they provide, what did you get out of them? (Please indicate N/A if this does not apply) (200 word count) (open text)
6. If you have already started or chosen a postdoctoral or other position, please complete the following: (text boxes)
    a. Institution and Department or other primary unit (such as Center or Institute)
    b. Name of primary mentor (PI)
7. How advanced are you in your planning for postdoctoral training after you finish your PhD? Please check the option that best describes your status for the following questions.
    a. How much effort have you made in communicating with potential postdoc mentors?
        i. None – have not contacted anyone
        ii. Contacted some – no serious discussions yet
        iii. Contacted some – started serious conversations
        iv. Have interviewed for at least one postdoc position
        v. Have a position lined up to start when I finish my PhD
        vi. Already in a postdoc position

b. On a scale from 1-5 how satisfied are you with:
   (1 being very unsatisfied, 5 being very satisfied)
   i. The quantity of guidance/help you received from your mentor
   ii. The quality of guidance/help you received from your mentor
   iii. The quantity of guidance/help you received from your other faculty
   iv. The quality of guidance/help you received from your other faculty

8. Several of last year's Academy sessions focused on careful planning of an academic career, beginning with choosing and/or designing your postdoctoral position. Did/are any specific elements from last year's sessions inform/ing your plans/search for a postdoc? If so, please describe. (200 word count) (open text)

9. How strong would you rate your skills in comparison to your peers in each of the following areas:

1 = quite a bit below my peers
2 = a bit below my peers
3 = very similar to my peers
4 = a bit above my peers
5 = quite a bit above my peers
NC = no clue – you really aren't sure how you compare to others

	Skill Level (1-5 scale above)
Skill	Now
Academic abilities (e.g. coursework)	
Research lab skills	
Research design skills	
Scientific thinking	
Scientific communication skills	
Networking	
Interpersonal skills	
Coping skills and stress reduction	

10. On a scale of 1-5 (1 being not helpful at all and 5 being extremely helpful), how much did each of these factors help you develop the skill?

Skill	Regular PhD program support	Sought out extra help or advice from PhD program (N/A – did not seek help/advice)	Support from the Academy
Academic abilities (e.g. coursework)			
Research lab skills			
Research design skills			
Scientific thinking			
Scientific communication skills			
Networking			
Interpersonal skills			
Coping skills and stress reduction			

11. When you think about the type of academic career you would like, what is the distribution of time you'd like to have?

    Predominantly teaching
    Predominantly research
    Roughly equal mixture of teaching and research
    Undecided
    If undecided, please briefly describe why (Comment Box)

12. On a scale of 1-10 (1 being lowest), please choose the number that corresponds to how you feel NOW about each of the following:
    a. How ACHIEVABLE does an academic career seem to you now?
    b. How DESIRABLE did an academic career seem to you now?

13. On a scale of 1-10 (1 being lowest), how CONFIDENT are you that you could:
    a. Obtain a faculty position in the future?
    b. Succeed in a faculty position once you obtain one?

14. On a scale from 0% (completely uncommitted) to 100% (completely committed), please identify how COMMITTED you are to an academic career RIGHT NOW? (Drop-down box 0-100)

15. One of last year's Academy sessions described the core elements of mentoring relationships. Did any of the elements from that presentation lead to any changes in your mentoring relationships, such as 'taking control of your own mentoring'? Did you become a more pro-active mentee in the last year? If so, please describe. (200 word count) (open text)

16. How useful did you find what you learned from each of the following on a scale of 1-10?
    a. Your time at the 2012 Summer Academy?
        i. What was most useful? (open text)
    b. From your coach?
        i. What was most useful? (open text)
    c. From your coaching group conference calls?
        i. What was most useful? (open text)
        ii. If you didn't participate, why not? (Check box w/multiple choices possible)
            1. Time not convenient
            2. Format not effective
            3. Topics not of interest
            4. Didn't connect with my group
            5. Didn't connect with my coach
            6. All the guidance I needed was provided by my PhD program
            7. Other (please specify-comment box)
    d. From other Academy participants?
        i. What was most useful? (open text)
    e. From Academy webinars?
        i. What was most useful? (open text)
        ii. If you did not participate in any, why not? (Check box w/multiple choices possible)
            1. Time not convenient
            2. Format not effective
            3. Topics not of interest
            4. Topics covered by my PhD program

    5. Other (please specify) (open text)

17. At the Academy, we talked about 3 different social science theories. Thinking back over the last year, do you recall ever remembering these theories and situations where you actually thought about them and they influenced your ways of thinking about things?
    a. **Cultural Capital:** Cultural Capital can be understood as *skills, knowledge, and ways of being* that can assist individuals to be successful scientists. People in power (such as your PI) promote those who act, look, and sound 'like them.'
    b. **Identity:** We all develop multiple identities as we grow up from an array of internal and external domains and experiences. Identity can be compromised when we don't experience people who 'look like us' with regard to a particular identity – literally and figuratively.
    c. **Communities of Practice:** A Communities of Practice perspective focuses especially on the ways groups use perceived competence with particular practices as a way to include or exclude certain individuals. The more a new member is different from current members of a C of P, the greater the chance they can be inadvertently marginalized and never achieve full insider status unless the C of P is actively open to what new members can bring.

18. How many times were you in contact (e.g. phone, email, or videoconference) with your coach over the course of this past year after the Academy meeting in July 2012? (Drop-down box 1-25+)

19. Outside of the periodic coaching group conference calls, how many times were you in contact with other Academy students after the Academy meeting in July 2012? (Drop-down box 1-25+)

20. Who from the Academy, both in your coaching group and outside of your coaching group, have you been in contact with outside of monthly group meetings?

21. After leaving last summer's Academy, did you consult the individual development plan you created? (Y/N)
    a. If yes, in what ways was it useful?
    b. If no, why not?

22. What are you hoping to get out of the upcoming summer Academy meeting? (open text)

23. What are you hoping to get out of the Academy during the next academic year? (open text)

24. Did you share any materials from the Academy with other students outside of the Academy? (Y/N)
    a. If so, which materials? Did they comment on if they used them? (open text)

25. Please provide any other comments about your experience(s) during the previous year of graduate school and/or the Academy not covered in the above that you would like us to know.
