# Supplementary material for: The Academy for Future Science Faculty: randomized controlled trial of theory-driven coaching to shape development and diversity of early-career scientists
Source: BMC Med Educ. 2014 Aug 2;14:160. doi: 10.1186/1472-6920-14-160 (PMC4121509; doi:10.1186/1472-6920-14-160)
Supplement: Additional file 6 — Interview protocol for The Academy for Future Science Faculty I and II Coaches in Winder 2014. [file 1472-6920-14-160-S6.pdf]

Research Study: The Academy for Future Science Faculty
Interview Questions for Academy I+II Coaches – Winter 2014

Rick McGee, PhD – Principal Investigator
Northwestern University, Feinberg School of Medicine
r-mcgee@northwestern.edu 312-503-1737

NORTHWESTERN UNIVERSITY

For the questions below, we're interested in hearing about your experiences and perspectives since your interview last fall.

1. How are things going with your coaching group? Have you had any group meetings or one-on-ones?
2. How engaged has your coaching group been? Have you noticed any differences in certain student's involvement? What is driving or limiting engagement? Do you see a difference in how engaged they were during the Academy meeting versus online/virtually?
3. Have you implemented any new technology or tools? If not, are you considering any new approaches to engage your group?
4. About how much time are you spending on Academy-related task? Are you satisfied with the amount of time? Was the amount of time just right, excessive, or less than you are willing to do?
5. Any issues within your group these last few months? Major successes or challenges?
6. Tell me about particular challenges you faced coaching your group, how you approached it, and whether or not it was successfully addressed.
7. Have the social science theories been useful for you since we spoke last fall? If so, how have you used them as an Academy coach? Have you used them in other aspects of your work?
8. Are there any ways in which you've started using what you've been learning through the Academy with students at your home institution?
9. When mentoring an Academy student, what are you doing that is similar or different than how you mentor or have mentored students in your own research group?
10. Do you have any suggestions for us as to how *The Academy for Future Science Faculty* might be improved, or how we might support your role as coach?
